# Development and Validation of a Simultaneous HPLC Method for Estimation of Bisoprolol Fumarate and Amlodipine Besylate from Tablets

**DOI:** 10.4103/0250-474X.44616

**Published:** 2008

**Authors:** D. N. Vora, A. A. Kadav

**Affiliations:** Chemistry Dept, Mithibai college of Arts, Chauhan Institute of Science and A. J. College of Commerce and Economics, Vile Parle (W), Mumbai-400 056, India

**Keywords:** Bisoprolol fumarate, amlodipine besylate, RP-HPLC

## Abstract

A fast, robust and stability indicating RP-HPLC method was developed for simultaneous determination of bisoprolol fumarate and amlodipine besylate in tablets. The mobile phase was mixture of 25 mM ammonium acetate adjusted to pH 5.0 and methanol (65: 35) at 0.8 ml/min. The stationary phase was Luna C18-2 column (3 μ, 50×4.6 mm ID). UV detection was performed at 230 nm. Retention time was 1.45 min and 3.91 min for bisoprolol and amlodipine, respectively. Linearity was established in the range of 8–33 μg/ml. Mean recovery was 99.1% and 98.6% for bisoprolol fumarate and amlodipine besylate, respectively.

Bisoprolol fumarate is a synthetic beta_1_ -selective cardioselective adrenoceptor blocking agent. The chemical name for bisoprolol fumarate is (±)-1-[4-[[2-(1-methylethoxy) ethoxy] methyl]phenoxyl-3-[(1-methylethyl)amino]-2-propanol(*E*)-2-butenedioate (2:1). It is a white crystalline powder, which is readily soluble in water, methanol, ethanol, and chloroform^1^. It is official in USP^2^.

Amlodipine besylate, a long-acting calcium channel blocker, is chemically described as 3-ethyl-5-methyl(±)-2-[(2-aminoethoxy)methyl]-4-(2-chlorophenyl)-1,4-dihydro-6-methyl-3,5-pyridine dicarboxylate, monobenzenesulphonate. Amlodipine besylate is a white crystalline powder. It is slightly soluble in water and sparingly soluble in ethanol^3^. It is official in BP^4^. Beta blocker plus calcium channel blocker combinations have utility in certain cardiovascular diseases like angina pectoris, myocardial infarction and hypertension. A tablet formulation containing bisoprolol fumarate and amlodipine besylate has been recently introduced on the market.

Various methods for determination of bisoprolol by fluorimetry^5^,^6^, HPLC^7^–^9^ and densitometry^10^–^12^ are reported in literature. Also HPTLC^13^–^16^, HPLC^17^–^22^, spectrophotometry 23–28 methods are reported for determination of amlodipine alone or in combination with other drugs. But, literature survey did not reveal any method for simultaneous determination of bisoprolol and amlodipine. The aim of this study was to develop a fast, precise, accurate, rugged and robust HPLC method for simultaneous determination of bisoprolol and amlodipine in tablets. Criteria employed for assessing suitability of proposed method was cost effectiveness and speed of analysis.

A liquid chromatographic system comprising of Waters 2695 separation module and Waters 2996 PDA detector (Waters Corporation, Milford, USA) connected to Empower chromatography software for processing the data generated were used. Reference standard of bisoprolol fumarate and amlodipine besylate was kindly supplied by Indoco Remedies along with certificate of analysis, and used as received. HPLC grade acetonitrile was purchased from J. T. Baker, NJ, USA, GR grade ammonium acetate was obtained from Merck, Mumbai, India and ExcelaR grade glacial acetic acid was supplied by Qualigens Fine Chemicals, Mumbai, India. The filter used in sample preparation was mdi SY25NN which was manufactured by Advanced Microdevices (P) Ltd, Ambala, India. The combination tablets containing bisoprolol fumarate and amlodipine besylate (Concor AM, Merck) were procured from the market.

A buffer solution was prepared by adjusting the pH of 25 mM ammonium acetate solution to 5.0 with acetic acid. The mobile phase was filtered and degassed mixture of buffer pH 5.0 and acetonitrile (65:35, v/v). Luna C18-2 column, (3 μ, 50×4.6 mm) was used as stationary phase. A constant flow of 0.8 ml/min was maintained throughout the analysis. Detection was carried out using PDA detector at 230 nm.

A combined standard stock solution of bisoprolol fumarate and amlodipine besylate was prepared in methanol (200 μg/ml). Five ml of standard stock solution was diluted to 50 ml with mobile phase to obtain a 20 μg/ml solution of bisoprolol and amlodipine and used as working standard for assay analysis. Twenty tablets were weighed and crushed to fine powder. An accurately weighed portion of the powder equivalent to 10 mg of bisoprolol fumarate and 10 mg of amlodipine was taken in 50 ml volumetric flask, about 30 ml of methanol was added to it and flask was kept in an ultrasonic bath for 2 min with intermittent swirling. This solution was then diluted to the mark with methanol and centrifuged. Five ml of the supernatant solution was diluted to 50 ml with mobile phase and mixed. This solution was filtered through mdi SY25NN filter and used for assay analysis. Five μl of each of working standard and sample solution were injected into the chromatograph and the peak areas were recorded. The amount of each active was computed by external standard quantification.

In order to optimize the LC separation of bisoprolol and amlodipine, initially, mobile phases of buffer and acetonitrile were used. The retention behavior of both the drugs was studied with respect to pH of buffer solution in the range of 3.0–6.8, and aqueous composition of mobile phase. Retention of both the drugs was found slightly dependant on pH of buffer (slight increase in retention with increase in pH). Bisoprolol was found relatively less sensitive to aqueous composition as against amlodipine, which was found more sensitive to aqueous composition. A ten percent increase in aqueous composition resulted in 1.6 and 3.3 times increase in retention for bisoprolol and amlodipine respectively. The buffer solution of pH 5.0 and mobile phase composition of buffer:acetonitrile (65:35) was found most appropriate for separation of bisoprolol and amlodipine on Luna C18-2 column. Flow rate was optimized based on capacity factor and column efficiency. Bisoprolol and amlodipine were well resolved in reasonable time of about 5 minutes. The retention times were 1.45 min and 3.91 min, respectively. The resolution between bisoprolol and amlodipine was 14.2. The final dilution of analytes with mobile phase helped to minimize the interference due to blank peaks. The wavelength of 230 nm was selected for the UV detection because at this wavelength there was maximum overlap of the spectra of bisoprolol and amlodipine. The peak purity of the peak due to bisoprolol and amlodipine was tested using PDA detector and were found to be pure.

To ascertain effectiveness of system suitability test, five replicate injections of freshly prepared working standard solution were injected into the chromatograph and relative standard deviation (RSD) of peak areas was calculated. The data is presented in Table 1. System suitability parameters such as tailing factor, resolution factor, capacity factor and theoretical plates of a typical chromatogram are tabulated in Table 2. Linearity (described by equation and corresponding corre-lation coefficient) was determined using five calibration levels for both the compounds (at 50-150% levels). The concentrations of calibration solutions of both the drugs were from 8 to 33 μg/ ml. The method of linear regression was used for data evaluation. Peak area of standard compounds was plot-ted against respective concentrations. The content of actives found in the commercial brand of tablets (Concor AM, Merck) by proposed method is shown in Table 1. The low values of RSD, indicates that method is precise. Intermediate precision was studied using different column, HPLC instrument and performing the analysis on different day. The results are presented in Table 1, along with repeatability data. Sample solution injected after 24 h of preparation did not show any appreciable change in assay value. To confirm the accuracy of the proposed method, recovery experiments were carried out by standard addition technique. Three different levels of standards were added to pre-analyzed tablet samples in triplicate. The mean percentage recoveries of bisoprolol and amlodipine were 99.1% and 98.6%, respectively. The results are shown in Table 1, which indicates that the method is accurate and precise and also there is no interference due to excipients present in the tablets. To ascertain the suitability of filter used for filtering sample preparation, the aliquot of sample solution was centrifuged and another aliquot of same sample solution was filtered through SY25NN filter. The percentage assay result of filtered sample was in close agreement with result of centrifuged sample, indicating that there was no adsorption of analytes on the filter. In order to evaluate specificity and stability indicating capability of the proposed method forced degradation studies were performed. The powdered samples of tablets were exposed to acidic, alkaline, strong oxidizing, heat and UV light conditions. Also, standard of bisoprolol and amlodipine were exposed to the above stress conditions, individually and in combination with each other to identify source of degradation peaks, if any. All the exposed standards and tablet samples were then analyzed by the proposed method. The results are given in Table 3. The assay values found lowered for bisoprolol in all the degradation conditions, however in case of amlodipine the assay values found lowered in all conditions except light. The assay values of both the ingredients were found decreased significantly in case of samples exposed to strong heat. Peaks due to bisoprolol and amlodipine in the chromatogram of all exposed samples were investigated using PDA detector and were found spectrally pure. The proposed method was subjected to robustness studies with respect to change in pH of buffer (±0.2 units), change in mobile phase composition (±3%), change in column temperature (35°) and change in flow rate (±0.1 ml). The results are presented in Table 2. The method was found robust with respect to variability in above conditions.

**TABLE 1:** METHOD VALIDATION RESULTS FOR INDIVIDUAL COMPOUND

Parameter	Bisoprolol	Amlodipine
System precision^a^ (% RSD)	0.5	0.8
Repeatability^b^ (% assay)	98.5	99.9
Repeatability^c^ (% RSD)	0.3	0.4
Intermediate precision^b^ (% assay)	99.5	99.9
Intermediate precision^c^ (% RSD)	0.5	0.4
Linearity^d^ (correlation coefficient)	0.99999	0.99999
Linearity^d^ (equation)	y = 8922.1×	y = 14126.9×
	- 1466.6	- 5056.6
Accuracy^e^ (% RSD)	0.8	0.4
Accuracy^e^ (% recovery)	99.1	98.6
Selectivity^f^	No	No
	interference	interference
Stability–ambient[%]^g^	99.5	98.7
Filter recovery^h^	99.8	99.6

a Determined on five replicate injections of working standard solution.

b Determined on six real samples of Concor AM tablets and average is reported.

c Percent RSD of six values of% assay of Concor AM tablets.

d Determined at five levels, from 50-150% of working standard concentration.

e Determined at three levels with triplicate determination at each level. Mean of 9 values and% RSD is reported.

f Demonstrated by forced degradation and peak purity of main peaks in degraded samples.

g Percent correlation of assay after 24 h of sample preparation against freshly prepared sample.

h Percent correlation of assay of filtered sample against assay of centrifuged sample.

**TABLE 2:** SYSTEM SUITABILITY PARAMETERS AND ROBUSTNESS

Component	Robustness parameter	k’^a^	T^b^	R^c^	N^d^	%Assay
Bisoprolol	No change (repeatability)	1.08	1.48	-	2596	98.5
	Organic in mobile phase (+3%)	1.20	1.38	-	1522	98.4
	Organic in mobile phase (-3%)	1.31	1.39	-	1928	98.8
	pH of buffer (+0.2 units)	1.18	1.50	-	2524	98.5
	pH of buffer (-0.2 units)	1.14	1.41	-	2770	98.5
	Column temperature 35°	1.31	1.39	-	3217	98.3
	Flow (+0.1 mL)	1.05	1.52	-	2733	98.1
	Flow (-0.1 mL)	1.20	1.43	-	2628	98.4
Amlodipine	No change (repeatability)	4.58	1.53	14.23	4985	99.9
	Organic in mobile phase (+3%)	3.79	1.54	9.23	3545	100.3
	Organic in mobile phase (-3%)	5.25	1.52	13.26	4618	99.7
	pH of buffer (+ 0.2 units)	4.53	1.52	13.24	4835	99.8
	pH of buffer (- 0.2 units)	4.42	1.50	13.71	5062	99.8
	Column temperature 35°	4.96	1.51	14.61	5614	99.4
	Flow (+ 0.1 mL)	3.92	1.50	12.16	4738	99.1
	Flow (- 0.1 mL)	4.29	1.55	12.65	4933	99.6

a Capacity factor determined for individual peak.

b Tailing factor determined for individual peak.

c Resolution factor determined between bisoprolol and amlodipine peaks.

d Column efficiency expressed as number of theoretical plates for bisoprolol and amlodipine peaks.

**TABLE 3:** FORCED DEGRADATION DATA

Degradation condition	Bisoprolol			Amlodipine		
		
	% Assay	Purity angle^a^	Purity threshold	% Assay	Purity angle^a^	Purity threshold
No degradation (Control)	98.5	1.177	1.598	99.9	0.480	1.278
Acid hydrolysis (1N HCl, 80°, 20 min)	95.3	1.191	1.357	94.1	0.380	0.981
Alkali hydrolysis (1N NaOH, 80°, 10 min)	95.4	0.725	1.421	93.9	0.406	0.942
Oxidation (30% H_2_O_2_, 80°, 30 min)	91.5	1.327	1.478	91.6	0.417	0.974
Thermal (105°, 1 d)	79.7	3.501	26.787	84.6	1.126	18.860
Photolytic (UV@254 nm, 1 d)	93.9	1.791	29.484	98.5	0.845	20.924

a For the peak to be pure, purity angle is required to be less than purity threshold.

The proposed method is fast, precise, accurate, rugged and robust for the simultaneous determination of bisoprolol and amlodipine from tablets. Hence it can be easily and conveniently adopted for the routine quality control analysis for assay as well as dissolution and content uniformity testing.
